# Impact of Screen Time on Language Development and Vocabulary Acquisition in Early Childhood: A Systematic Review

**DOI:** 10.7759/cureus.97429

**Published:** 2025-11-21

**Authors:** Evangeline C Nwachukwu, Aahana Nigam, Sandeep Sekar lakshmisai, Priyanka Sakarkar, Roshitha S Bheemaneni, Iana A Malasevskaia

**Affiliations:** 1 Department of Planning, Research, and Statistics, Nigeria Centre for Disease Control and Prevention, Abuja, NGA; 2 Department of Cardiology, Trinity College Dublin, Dublin, IRL; 3 Department of Medicine, SRM Prime Hospital, Chennai, IND; 4 Department of General Surgery, Princess Royal University Hospital, Orpington, GBR; 5 Department of Gastroenterology, AdventHealth Ocala, Ocala, USA; 6 Department of Internal Medicine, Gandhi Medical College, Hyderabad, IND; 7 Principles and Practice of Clinical Research, Harvard T.H. Chan School of Public Health, Boston, USA

**Keywords:** children, communication skills, digital media, early literacy, language development, preschool, screen exposure, screen time, smartphones, vocabulary acquisition

## Abstract

Language development in the first few years of life is critical for later academic and social success. With the increasing use of digital devices among preschoolers, there have been concerns about the potential impact of screen time on language outcomes. Existing research presents mixed findings, making it necessary to synthesize current evidence. This systematic review aimed to examine and synthesize empirical evidence on the relationship between screen time and language development in early childhood, with emphasis on factors such as device type, content quality, socioeconomic status, and parent-child interaction.

Following the Preferred Reporting Items for Systematic Reviews and Meta-Analyses (PRISMA) guidelines, a comprehensive search was conducted in the Cochrane Library, PubMed, Medical Literature Analysis and Retrieval System Online (MEDLINE), EBSCO Open Dissertations, ScienceDirect, and Clinicaltrials.gov for studies published between January 1, 2020, and February 17, 2025.

Our inclusion criteria required studies published in the English language, assessed screen time exposure, and measured language outcomes in typically developing preschoolers. Data were extracted using a standardized form. The risk of bias was assessed using the Cochrane Risk of Bias 2 (RoB 2), the Risk Of Bias In Non-Randomized Studies of Interventions (ROBINS-I), and the Joanna Briggs Institute (JBI) Critical Appraisal tools. The overall certainty of the evidence was evaluated using the Grading of Recommendations, Assessment, Development, and Evaluations (GRADE) framework.

A total of eight studies met the inclusion criteria, mostly consisting of cross-sectional studies that involved diverse patient populations with sample sizes ranging from 83 to 4,907 participants. The participants were primarily children aged three to six years and/or their parents. These studies mainly focused on measuring the duration of screen time, which averaged between approximately 1.39 and 2.65 hours per day. One study analyzed 44 mobile applications for their learning goals and educational potential. The most commonly reported outcomes were related to language development and vocabulary acquisition, which were assessed through parental surveys and developmental scales.

Synthesized evidence suggests that high levels of unsupervised or passive screen time are often linked to weaker language development outcomes in preschoolers. However, screen use that is interactive, educational, and involves caregiver participation appears to mitigate these potential effects. The overall certainty of this evidence, however, remains limited. Future research should prioritize consistent measurement approaches and explore long-term impacts.

## Introduction and background

Language acquisition is a key developmental milestone attained in early childhood [1]. By age five, children develop a vast vocabulary, use complex language, and apply these skills to reading; mature speech develops around age eight [1, 2]. Wittgenstein emphasized the link between language and cognitive development [3]. Regarding language acquisition, nativist theories propose that humans possess an inherent and biological capacity for language, while empiricists suggest that learning is primarily driven by environmental input. Contemporary research suggests an interconnected framework involving both [1]. Speech and language impairments are common among preschoolers [2], underscoring the importance of early language development and intervention to support communication skills.

"Screen time" refers to time spent exposed to electronic media [4, 5]. Early research has reported associations between excessive screen time and delayed language development. During critical periods of development, prolonged exposure has been associated with reduced social interactions and limited linguistic input, which may influence phonetic, lexical, and syntactic development. Face-to-face interactions are essential for language feedback [6]. The American Academy of Pediatrics (AAP) recommends avoiding screen use for children under 18 months (except for video chatting) and limiting it to under one hour daily for ages two to five, with shared viewing and avoiding screens during meals and bedtime [7, 8]. The World Health Organization (WHO) provides similar recommendations [9].

Previously, home television viewing was predominant, but today's children have access to a diverse range of screen-based devices [10, 11]. This has transformed screen time habits, including solitary viewing, multitasking, and increased use in educational settings. Infants are now exposed to screens at increasingly younger ages, contributing to increased screen media consumption [10-12]. Some studies show average daily screen time ranging from 3.6 to four hours [13, 14]. Kucker et al. [6] reported that children under 30 months spent twice as much time watching TV/videos as reading books. The COVID-19 pandemic further accelerated screen use [15, 16]. This was further reinforced by Fitzpatrick et al. [15], who found that during the pandemic, 64% of preschoolers exceeded recommended screen time limits.

Reports on the effects of screen time on language development vary. Whereas earlier research linked excessive screen time to fewer language opportunities [11, 13, 17], recent studies suggest that the effects are complex, influenced by culture, personal preferences, and context [6]. While excessive passive exposure may hinder language skills, varied approaches with educational content and parental involvement may benefit children [11, 16]. The rapidly evolving landscape of screen exposure raises questions about how technology shapes interactions, perceptions, and language skills [8, 11].

Prior research on screen time primarily focused on older children, leaving a gap concerning younger children's critical brain development [10, 17]. This systematic review compiles and synthesizes evidence on how screen time affects early childhood language development, acknowledging the evolution of screen modalities. The review aims to inform parents, educators, and policymakers about the potential benefits and risks associated with screen time exposure during the formative years.

## Review

Methods

Study Design

This systematic review was conducted following the Preferred Reporting Items for Systematic Reviews and Meta-Analyses (PRISMA) 2020 guidelines to ensure transparency and rigor in the review process [18]. The aim was to evaluate the impact of screen time on language development and vocabulary acquisition in early childhood. This review was not registered on the International Prospective Register of Systematic Reviews (PROSPERO).

Population, Intervention, Comparison, Outcome, Type of Studies​​​​​​ (PICOT) Framework

The PICOT framework was utilized to structure the systematic review, focusing on specific elements critical to understanding the relationship between screen time and language development in early childhood. The population (P) of interest consisted of children aged 6 years or younger. This age range was selected to capture the developmental stage where language acquisition is most crucial and sensitive to environmental influences, such as screen exposure. There was no separate analysis for children aged two years and below. The intervention (I) examined was screen time. For the comparison (C) group, studies that assessed the impact of screen time on language development and vocabulary acquisition were eligible for inclusion even if they did not include a comparative group. This approach allowed for a broader range of evidence to be considered, capturing various perspectives on the relationship between screen time and language outcomes in early childhood. As a result, both comparative and non-comparative studies contributed to the overall understanding of the topic.

The primary outcome (O) measured was the level of language development and vocabulary acquisition in children subjected to different amounts of screen time. This outcome was chosen to assess how varying levels of screen exposure might influence critical language skills during formative years. The type of studies (T) included in this review comprised observational studies, randomized controlled trials (RCTs), non-randomized clinical trials, and qualitative studies published within the last five years, ensuring a contemporary understanding of the topic.

Inclusion and Exclusion Criteria

Studies were included in the review based on specific criteria. Only research that aligned with the PICOT framework was considered. Additionally, studies needed to focus on children aged six years or younger to ensure relevance to the target population. The inclusion of studies involving infants younger than 18 months does not endorse screen use at this age, but captures real-world practices that diverge from existing AAP and WHO recommendations. Only research published in the English language within the last five years was included, allowing for a focus on the most recent findings and methodologies in the field. Grey literature (e.g., dissertations) and non-participant application (app)-evaluation studies were included only if they provided empirical data relevant to screen time or language-related outcomes.

Conversely, several exclusion criteria were established to maintain the quality and relevance of the review. Studies that were non-original or had incomplete data or results were excluded to avoid bias and ensure comprehensive analysis. Research involving children older than six years was also excluded, as the developmental focus was specifically on early childhood. Furthermore, studies that did not report on screen time or language development were omitted, as they would not contribute to answering the research question. In addition, studies not published in English were omitted from this review. Finally, any studies assessed as having a high risk of bias or poor methodological quality based on standardized tools were excluded to ensure that only reliable evidence informed the review's conclusions.

*Search* *Strategy*

A comprehensive search was conducted from February 6^th ^to February 17^th^, 2025, across multiple databases, including Cochrane Central Register of Controlled Trials (CENTRAL), PubMed/Medical Literature Analysis and Retrieval System Online (MEDLINE), ScienceDirect, EBSCO Open Dissertations, and ClinicalTrials.gov. The search strategy employed a combination of keywords and MeSH terms related to screen time, language development, and vocabulary acquisition, tailored to the specific databases and registers. Search filters were applied to restrict results to studies published in English within the last five years. The complete database search strings used are presented verbatim in Table 1, along with the number of records retrieved and retained before and after applying inclusion and exclusion criteria. This transparent reporting aligns with PRISMA 2020 recommendations for reproducibility.

**Table 1:** Search Strategy Total after initial search: 5359; Total after application of inclusion/exclusion criteria: 337

Databases	Search Strategy	Filters used	Number of studies before/after I/E criteria
Cochrane Central Register of Controlled Trials (CENTRAL)	#1 "Screen time" OR technology OR “electronic device” OR tablets OR smartphones OR "media use" OR television 83123 #2 MeSH descriptor: [Screen Time] explode all trees 84 #3 #1 OR #2 83123 #4 "Language development" OR "speech development" OR "speech production" OR "communication skills" OR "early literacy" OR "expressive language" 4889 #5 MeSH descriptor: [Language Development] explode all trees 926 #6 #4 OR #5 5370 #7 Child OR children 219877 #8 MeSH descriptor: [Child] explode all trees 83857 #9 #7 OR #8 219877 #10 #3 AND #6 AND #9 209	Trials, last 5 years, English	209/47
PubMed/ Medical Literature Analysis and Retrieval System Online (MEDLINE)	("Screen time" OR technology OR tablets OR smartphones OR "digital media" OR television OR “Social media use” OR “recreational screen media use” OR touchscreen OR “television viewing time” OR “screen exposure” OR “mobile technology devices” OR "Screen Time"[Mesh]) AND ("Language development" OR "speech development" OR "speech production" OR "communication skills" OR "early literacy" OR "expressive language" OR speech OR “language skills” OR “cognitive development” OR "Language Development"[Mesh]) AND (Child* OR “early childhood” OR preschool OR “young children” OR "Child"[Mesh])	In the last 5 years, Full text, Adaptive Clinical Trial, Case Reports, Classical Article, Clinical Study, Clinical Trial, Comparative Study, Controlled Clinical Trial, Multicenter Study, Observational Study, Randomized Controlled Trial, English, Humans	4487/166
ScienceDirect	("Screen time" OR “screen exposure” OR “mobile technology devices”) AND ("Language development" OR "speech development" OR "communication skills" OR "early literacy" OR “vocabulary acquisition”) AND (young children)	last 5 years, research articles, case reports, short communications, English, open access	349/70
EBSCO Open Dissertations	("Screen time" OR technology OR tablets OR smartphones OR "digital media" OR television OR “Social media use” OR touchscreen OR “television viewing time” OR “screen exposure” OR “mobile technology devices”) AND ("Language development" OR "speech development" OR "speech production" OR “vocabulary acquisition” OR "communication skills" OR "early literacy" OR "expressive language" OR speech OR “language skills” OR “cognitive development”) AND (Child* OR “early childhood” OR preschool OR “young children”)	Last 5 years, Dissertations	208/51
Clinical trials.gov	"Language development" OR "speech development" OR "speech production" OR "communication skills" OR "early literacy" OR "expressive language" OR "speech" OR “language skills” | "Screen time" OR technology OR tablets OR smartphones OR "digital media" OR television OR “Social media use” OR “recreational screen media use” OR touchscreen OR “television viewing time” OR “screen exposure” OR “mobile technology devices” |	Completed studies| Child (birth - 17) | Interventional, Observational studies| studies with results| study completion from 01/01/2020 to 02/06/2025	106/3

Screening and Data Management

The Rayyan app (Rayyan Systems Inc., Cambridge, MA) was utilized for the screening process to facilitate the removal of duplicates and the assessment of study eligibility [19]. Two independent reviewers screened the titles and abstracts of identified studies, and discrepancies were resolved through discussion and consensus.

Data Extraction

From the eight studies included in this review, data were extracted using a standardized extraction form that captured relevant information such as study design, population characteristics, intervention details, outcome measures, and key findings. This systematic approach ensured comprehensive data collection for subsequent analysis and synthesis.

Quality Assessment

The methodological quality of the included studies was assessed using critical appraisal tools appropriate for each study design. This assessment was conducted independently by two reviewers to evaluate the risk of bias and the overall quality of evidence.

The Cochrane Risk of Bias 2 (RoB 2) tool was employed to assess the risk of bias in RCTs [20]. It evaluated five key domains: 1. bias arising from the randomization process, 2. bias due to deviations from intended interventions, 3. bias due to missing outcome data, 4. bias in measurement of the outcome, and 5. bias in the selection of the reported result(s). Each domain was judged as either low risk, some concerns, or high risk. In this review, only studies rated as having low risk or some concerns were included, while those with high risk of bias were excluded to maintain internal validity.

For non-randomized clinical trials, the Risk Of Bias In Non-Randomized Studies - of Interventions (ROBINS-I) tool was utilized [21]. This tool assessed seven domains that included confounding, selection of participants, classification of interventions, deviations from intended interventions, missing data, measurement of outcomes, and selection of reported results. Similar to RoB 2, studies with low or moderate risk of bias were included, while those with high risk were excluded to enhance the reliability of the findings.

The Joanna Briggs Institute (JBI) Critical Appraisal Tool for Cross-Sectional Studies was used to evaluate methodological quality through eight specific questions, including inclusion clarity, sample description, valid exposure measurement, objective and valid outcome measurement, identification and control of confounders, and appropriate statistical analysis [22]. Each of the eight checklist items was rated “yes,” “no,” or “unclear.” Studies scoring at least 6 of 8 (“yes”) were considered methodologically adequate for inclusion, ensuring that only studies of acceptable quality were considered in the review.

The outcome of these appraisals is summarized in the result section, which presents domain-specific ratings and overall judgments for each included study. This stepwise evaluation ensured that only evidence of acceptable methodological rigor contributed to the synthesis.

*Assessment of the Certainty of Evidence* 

The overall certainty of the evidence for the key outcomes was assessed using the Grading of Recommendations, Assessment, Development, and Evaluations (GRADE) framework. The certainty was judged for the following predefined outcomes: language development, vocabulary acquisition, and the impact of interactive versus passive media. The evidence from randomized trials started as high certainty, and from observational studies as low certainty. The certainty was then downgraded based on five domains: risk of bias, inconsistency, indirectness, imprecision, and publication bias. Two reviewers independently made these judgments, and any discrepancies were resolved through consensus. The final certainty ratings were categorized as high, moderate, low, or very low.

Data Synthesis

Data from the included studies were presented narratively to summarize the findings and provide an overview of how screen time influences language development and vocabulary acquisition in early childhood. They were also organized into tables and charts for a clear and organized presentation of the findings, making it easier to compare results across different studies.

Results

**Figure 1:**
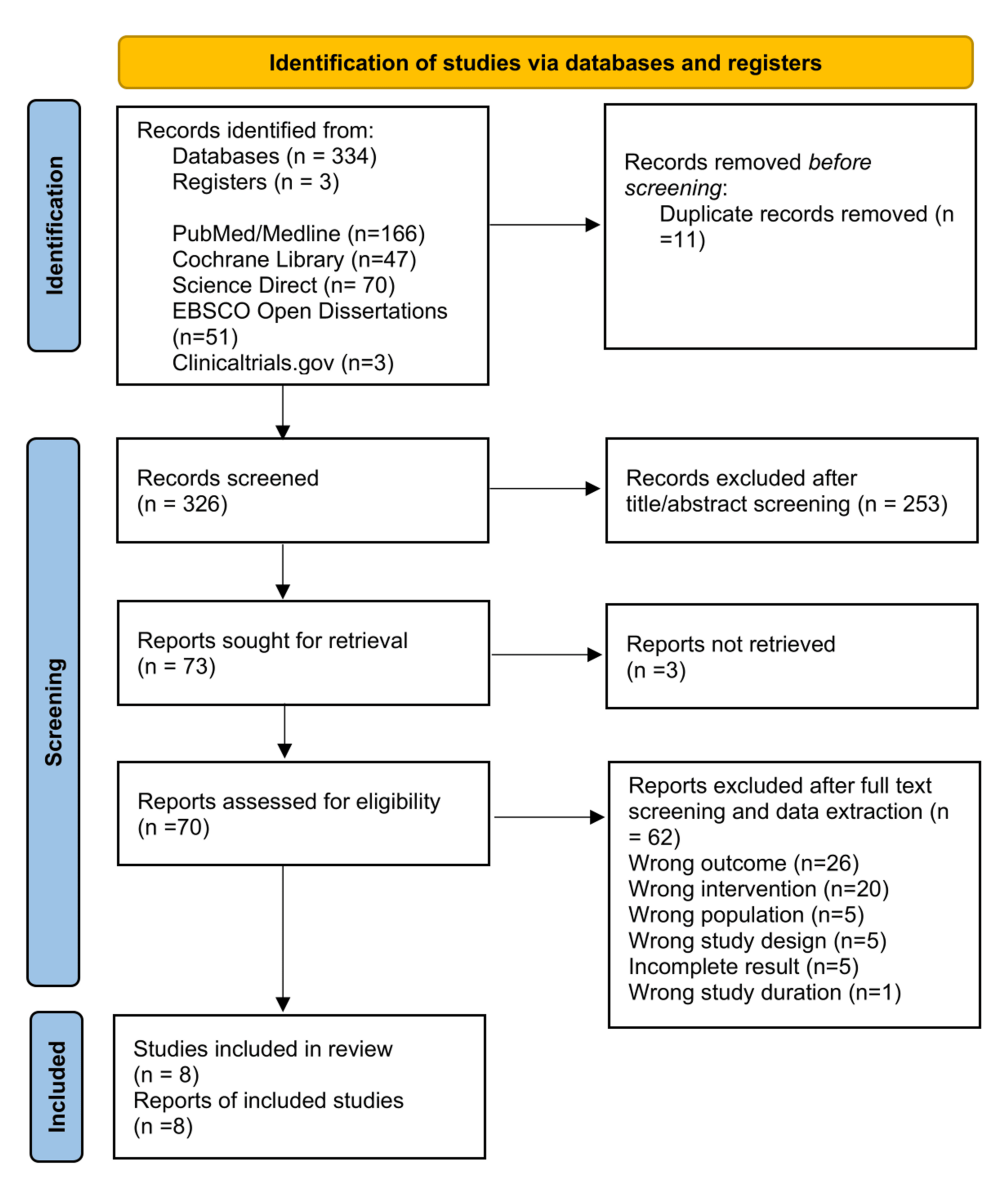
PRISMA flow diagram PRISMA: Preferred Reporting Items for Systematic Reviews and Meta-Analyses; MEDLINE: Medical Literature Analysis and Retrieval System Online

A total of 5,359 records were identified across four databases and one register following the initial search. Upon application of the inclusion and exclusion criteria, 337 records (334 from databases and three from registers) remained. The references were subsequently imported into Rayyan [19], resulting in the removal of 11 duplicates. Of the 326 records assessed for title/abstract eligibility, 73 studies were selected for full-text evaluation. Ultimately, eight studies fulfilled the eligibility criteria and underwent a quality appraisal, leading to their inclusion in this review. These stages are detailed in the PRISMA 2020 flow diagram (Figure 1). This structured and multi-database approach ensured comprehensive coverage of relevant literature and aligns with the PRISMA 2020 reporting standards [18].

Quality Assessment

In this systematic review, only studies classified as having "some concerns" or "low risk of bias" according to the Cochrane RoB 2 tool were included [23]. Furthermore, studies that exhibited low or moderate risk of bias on the ROBINS-I tool were considered eligible [24], as well as cross-sectional studies that attained a minimum score of 6 out of 8 on the JBI Critical Appraisal Tool for Cross-Sectional Studies [22].

Table 2 presents the quality assessment for one RCT using the Cochrane RoB 2 Tool [20]. This study demonstrated low risk and was included in the review.

**Table 2:** Quality appraisal for RCTs Cochrane RoB 2 tool assesses five domains: Domain 1 (bias arising from the randomization process), Domain 2 (bias due to deviations from intended interventions), Domain 3 (bias due to missing outcome data), Domain 4 (bias in measurement of the outcome), and Domain 5 (bias in selection of the reported result(s)) Cochrane RoB 2: Cochrane Risk of Bias 2; RCT: randomized controlled trial

Study	Domain 1	Domain 2	Domain 3	Domain 4	Domain 5	Overall
Guevera et al. [23]	Low risk	Low risk	Low risk	Low risk	Low risk	Low risk

Table 3 presents the quality assessment for one non-randomized clinical trial using the ROBINS-I tool [21]. This study demonstrated a moderate risk and was included in the review.

**Table 3:** Quality assessment for one non-randomized clinical trial using the Risk of Bias In Non-Randomized Studies - of Interventions tool Domain 1: Risk of bias due to confounding, Domain 2: Risk of bias in classification of interventions, Domain 3: Risk of bias in selection of participants into the study (or into the analysis), Domain 4: Risk of bias due to deviations from intended interventions, Domain 5: Risk of bias due to missing data, Domain 6: Risk of bias arising from measurement of the outcome, Domain 7: Risk of bias in selection of the reported result

Study	Domain 1	Domain 2	Domain 3	Domain 4	Domain 5	Domain 6	Domain 7	Overall
Taylor et al. [24]	Moderate risk	Low risk	Low risk	Low risk	Low risk	Low risk	Low risk	Moderate risk

Table 4 summarizes the risk of bias assessment for six cross-sectional studies included in this review, evaluated using the JBI Critical Appraisal Checklist for Analytical Cross-Sectional Studies [22].

**Table 4:** Quality appraisal of cross-sectional studies using JBI Critical Appraisal Checklist for Analytical Cross-Sectional Studies JBI: Joanna Briggs Institute

	Chen et al. [25]	Turco [26]	Gath et al. [27]	Rai et al. [28]	Chaibal et al. [29]	Nobre et al. [30]
Q 1. Were the criteria for inclusion in the sample clearly defined?	Yes	Yes	Yes	Yes	Yes	Yes
Q2. Were the study subjects and the setting described in detail?	Yes	Yes	Yes	Yes	Yes	Yes
Q 3. Was the exposure measured in a valid and reliable way?	Yes	Yes	Yes	Yes	Yes	Yes
Q 4. Were objective, standard criteria used for measurement of	Yes	Yes	Yes	Yes	Yes	Yes
Q 5. Were confounding factors identified?	Yes	Yes	Yes	Yes	Yes	Yes
Q 6. Were strategies to deal with confounding factors stated?	Yes	Yes	Yes	Yes	Yes	Yes
Q7. Were the outcomes measured in a valid and reliable way?	Yes	Yes	Yes	Yes	Yes	Yes
Q 8. Was appropriate statistical analysis used?	Yes	Yes	Yes	Yes	Yes	Yes

Summary of the Included Studies

This review included a total of eight studies that examined the relationship between screen time and language skills in early childhood. The studies employed a range of designs, thereby providing a comprehensive view of the topic. The studies spanned a variety of populations, with participants primarily consisting of children aged between three to six years.

Among the included studies, the most frequently reported outcomes were language development and vocabulary acquisition, assessed through various measures, including the Bayley Scales of Infant and Toddler Development (Bayley-III).

The most frequently reported outcomes were language development and vocabulary acquisition, assessed through measures like parental surveys and developmental scales. The studies primarily measured the duration of screen time, with average usage ranging from approximately 1.39 to 2.65 hours per day. Factors such as the quality of content consumed (educational vs. non-educational) and parental engagement during screen activities were also assessed, with many studies highlighting the importance of high-quality interactions.

Overall, the findings suggest that while screen time can have mixed effects on language development, the quality of content and the nature of parental involvement are critical determinants of outcomes (Table 5).

**Table 5:** Summary of the included studies App: application; LG: learning goal; NLG: no learning goal; MLU: mean length of utterance; AoA: age of acquisition; SES: socioeconomic status; DLP: digital literacy promotion; SLP: standard literacy promotion; CELF-P2: Clinical Evaluation of Language Fundamentals: Preschool, Second Edition; TV: television; PARCHISY: Parent-child Interaction System

Study	Sample size/Population characteristics	Intervention/Exposure	Outcome measures	Key findings	Notes
Guevara et al. [23]	104 Medicaid-eligible infants (five to six months old); diverse backgrounds from urban, predominantly Black to suburban populations, predominantly White populations.	DLP: Enhanced e-books on home digital devices during well visits from six to 12 months of age. SLP: Board books provided at well visits from six to 12 months of age.	StimQ-Read Subscale scores parent-reported reading and television use. Bayley Scales of Infant Development -3rd Edition (Bayley-3) scores.	SLP had higher StimQ-Read scores at seven to eight months (9.5 vs. 7.5, P = .011). DLP parents reported more digital reading (65% vs. 23%, P < .001). Slightly lower language scores in DLP (85.7 vs. 89.7).	E-books are feasible in practice. Possible adverse effects on language development.
						Taylor et al. [24]	Total apps analyzed: 44 apps. Apps with an LG: 18. Apps with NLG: 26. App selection criteria: Top 10 free and paid apps for preschool children from Apple, Google, and Amazon app stores.	Use of touchscreen apps was categorized by the presence of a learning goal. Each app was analyzed for a five-minute usage session.	Educational Potential Index: Score based on 10 items (0-2 points each). Psycholinguistic Measures: MLU, frequency of single/multi-word utterances, word frequency, concreteness, and age of acquisition. Feedback Types: Proportion of ostensive feedback vs. non-specific feedback.	Apps with learning goals scored higher in educational potential (10.83 vs. 4.04, p < .001). More ostensive feedback and language content in LG apps. MLU was slightly lower in LG apps (4.58 vs. 5.22).	LG apps create meaningful learning. Recommendations: encourage exploration, improve content/social use. Small sample; limited app selection.
												Chen et al. [25]	Total parents surveyed: 4,907; Location: Medium-to-low SES community in a first-tier city in China; Demographics: 80.4% mothers, Children's age range: three to six years (Mean age = 49.64 months), 52.90% boys, 36.2% single-child Families	Devices analyzed: TVs, tablets (including smartphones), computers, and paper-based books. Focus: Investigated the impact of these devices on parental efficacy and home literacy practices.	Device exposure variables: Frequency of usage for TV, tablets, computers, and the number of books at home. Home literacy practice variables: Frequency of reading and storytelling by parents, and child self-reading.	TVs negatively impacted literacy (b = –0.080). Tablets negatively affected storytelling (b = –0.050). Computers positively correlated with literacy (b = 0.061). High parental efficacy was linked to more books & less screen use.	Low-efficacy parents relied on screens. SES shaped access and practices. Findings are culturally specific (China).
																		Turco [26]	The sample included 881 four- and five-year-olds who had completed at least one language and literacy assessment in the second year and whose parents completed the home environment questionnaires	Engagement with educational content on mobile devices (e-books, apps)	Quality interactions: Assessed through observations of parent-child engagement during digital book reading. Parent Self-Efficacy: Evaluated through parental confidence in supporting their child’s learning with mobile media. Frequency of Reading Requests: Measured how often children asked to be read to. Device Usage: Recorded how children interacted with various devices (e.g., time spent on e-books vs. games).	Vocabulary ↑ by 15%. 70% improved phonemic awareness. 75% of interactions were high-quality. Parents with high self-efficacy had 50% better interaction quality.	Kids prefer games/videos over apps. Apps need design improvements for engagement.
						Gath et al. [27]	84 parents and their preschoolers (ages three to five years); diverse ethnic backgrounds, with 71.4% New Zealand European.	Screen time (television and electronic media). Average daily screen time- 1.78 hours (weekdays), 2.65 hours (weekends).	Language production and comprehension (CELF-P2), Parent-child relationship (Child-Parent Relationship Scale).	TV negatively correlated with shared reading (r = 0.68). Electronic media negatively correlated with language production (r = –0.43). Screen time reduces parent-child closeness.	Supports the displacement hypothesis. Suggests reducing screen time to promote bonding/language.												
																								Rai et al. [28]	44 parent-child pairs, Mean age: 3.5 years (SD = 0.3)	Screen time activities: Watching videos, playing electronic games, reading storybooks; Duration: 25-30 min per session, with a 2-week daily diary for screen time use	Cognitive development assessed via: Working memory, inhibitory control, vocabulary, self-control; Parent-child interaction quality measured using the PARCHISY scale	Screen time negatively correlated with working memory (rₛ = –0.40). Educational screen time positively correlated with vocabulary (rₛ = 0.38). Interaction quality is highest in games; lowest in video viewing.	Reliable results. Content quality matters more than duration.
Chaibal et al. [29]	85 community children (mean age: 4.05 ± 0.91 years)	Duration of smartphone and tablet usage recorded over seven days; Average usage: 82.78 ± 62.82 min/day.	Developmental aspects assessed: gross motor, fine motor- adaptive, language, and personal- social skills.	No significant correlation with language. Positive correlation with gross motor (p = .036). Caregiver use linked to child screen use.	33% showed suspected fine motor delays. SES affected personal-social outcomes.																								
																		Nobre et al. [30]	103 children aged 24 to 42 months	Interactive media usage (smartphones and tablets)	Cognitive Development: Assessed using the Bayley Scale of Infant and Toddler Development. Expressive language, fine motor development, and gross motor development were assessed using the Bayley Scale.	The quality of interactive use correlated with language (r = 0.40). Interactive media explained 20% of language variance. Increased vocabulary and literacy with interactive use.	Smartphones are used more than tablets (median 45 minutes/day). Interactive media fosters engagement unlike passive TV.						

The analysis of screen exposure reveals varied impacts on developmental outcomes in children. High television usage consistently correlates with poorer literacy practices and language engagement. In contrast, computer use tends to correlate positively with literacy development, suggesting that interactive content may enhance these skills.

Interactive media, such as smartphones and tablets, are generally associated with favorable language outcomes. High-quality interactive experiences can foster vocabulary growth and improve language skills. However, concerns arise regarding digital reading formats, which have been associated with lower language engagement in some studies. 

Conversely, excessive screen time, particularly from television, negatively correlates with shared reading practices and language production. This indicates that passive screen exposure may displace meaningful literacy activities. Overall, while interactive and educational media can enhance vocabulary and literacy, passive screen time often has detrimental effects on language development. 

Grey Literature and App-Based Studies

Among the included records, one study represented grey literature (a doctoral dissertation [26]), and one was an app-evaluation study that did not involve direct participants [24]. These were analyzed separately from the primary empirical studies because they contribute contextual rather than outcome-level evidence. The dissertation provided observational insights into parent-child interactions with mobile screen media, while the app study evaluated the linguistic content and learning potential of preschool applications. Although both sources enhance understanding of the broader digital media environment, their findings were interpreted cautiously and not weighted equally with primary data in the synthesis.

Results of the GRADE Assessment 

Based on the GRADE assessment of the evidence regarding the impact of screen media exposure compared to traditional book reading on language development and home literacy practices in children aged between 0 and six years, the overall quality of evidence is very low to moderate. Key findings indicate that the relationship between screen use and language outcomes is highly context-dependent. For instance, the RCT study provided moderate-quality evidence that e-books were associated with slightly lower language scores compared to traditional board books, though the study was underpowered. Similarly, evidence on vocabulary acquisition and general language development was predominantly of very low quality due to serious risks of bias, inconsistency across studies, and imprecision (Table 6). However, low-quality evidence suggests that interactive media and active parental involvement during screen use may mitigate negative effects and support language development. These findings highlight the need for higher-quality, longitudinal studies to clarify the role of screen media in early language and literacy environments.

**Table 6:** GRADE Summary of Findings (SoF) Research question: What is the impact of screen media exposure (including e-books) versus traditional book reading on language development and home literacy practices in children aged between 0 and six years? Population: Children aged between 0 and six years; Intervention: Screen media use (e-books, tablets, TV, computers); Comparison: Traditional book reading (board books, print books) with or without parent interaction GRADE: Grading of Recommendations, Assessment, Development, and Evaluations; RCT: randomized controlled trial

Outcome	No. of Studies (Design)	Risk of Bias	Inconsistency	Indirectness	Imprecision	Overall Quality of Evidence (GRADE)	Comments and Findings
Language development (Overall)	9 studies (2 RCTs, 1 non-randomized trial, 6 observational)	Serious ⚠️	Serious ⚠️	Not serious	Serious ⚠️	Very Low 🔻🔻🔻◯	Conflicting results; some show negative effects of e-books, others show no difference.
Vocabulary acquisition	5 studies (observational)	Serious ⚠️	Serious ⚠️	Not serious	Serious ⚠️	Very Low 🔻🔻🔻◯	Parent-reported measures: mixed results.
Screen time vs. Language development correlation	8 studies (observational)	Serious ⚠️	Serious ⚠️	Not serious	Serious ⚠️	Very Low 🔻🔻🔻◯	Direction of effects inconsistent; confounding likely.
Interactive vs. Passive media effects on language	4 studies (1 RCT, 3 observational)	Serious ⚠️	Not serious	Not serious	Serious ⚠️	Low 🔻🔻◯◯	Interactive media may support language, but on small samples.
Parental mediation during reading	5 studies (1 RCT, 4 observational)	Serious ⚠️	Not serious	Not serious	Serious ⚠️	Low 🔻🔻◯◯	Parental involvement improves outcomes; consistent but limited evidence.
E-books vs. Board books: Language outcomes	1 RCT (Guevara et al., 2021 [23])	Not serious	Not serious	Not serious	Serious ⚠️	Moderate 🔻🔻🔻◯	E-books associated with lower language scores (−4 points, p=0.10); underpowered.
E-books vs. Board books: Home literacy environment	1 RCT (Guevara et al., 2021 [23])	Not serious	Not serious	Not serious	Serious ⚠️	Moderate 🔻🔻🔻◯	No significant difference in StimQ-Read scores; initial advantage for board books.

Discussion

This systematic review aimed to assess the impact of screen time on language development and vocabulary acquisition in early childhood, a period considered critical for linguistic and cognitive growth. This review also incorporates newer forms of screen use and screen-based devices and underscores the complex and nuanced relationship between screen exposure and language outcomes by synthesizing evidence from eight diverse studies. While this review included one dissertation [26] and one app-based evaluation [24], these were treated as contextual evidence rather than primary empirical studies. Their inclusion expands the conceptual understanding of digital media quality but does not directly inform causal or correlational inferences about child language outcomes. This distinction helps maintain the methodological integrity of the evidence synthesis.

Our first main finding shows that screen exposure was common among young children, with television being the most frequently used device, especially among preschoolers [25-27]. The findings suggest that while screen time, mainly passive viewing, has been linked to poorer language outcomes, the interactive nature of the device, the quality of content, the age of the child, the nature of interactions, socioeconomic status (SES), and parental involvement significantly mediate these effects [23-30].

However, it is critical to contextualize these findings within the overall low certainty of the evidence, as assessed by the GRADE framework. For most outcomes, including overall language development and vocabulary acquisition, the evidence was graded as very low to low certainty. This was primarily due to the preponderance of observational study designs, which carry a serious risk of confounding bias, as well as inconsistency in results and imprecision in effect estimates. Consequently, while the synthesized results provide important insights for hypothesis generation and clinical guidance, the true effect of screen media on language development may be substantially different. This underscores the need for cautious interpretation and highlights the necessity for more rigorous, high-quality primary studies.

Nature of the Device

Televisions were typically associated with passive screen viewing and represented the majority of the screen devices used among young children [27]. This form of engagement often displaces developmentally enriching activities, such as physical play and interactive communication with caregivers [25, 27]. Several studies noted that although children spent considerable time watching TV, up to 88 minutes per day, only a small portion was devoted to educational programming [25].

Tablets, smartphones, and computers also featured prominently in children's screen use, providing both passive content (e.g., videos, cartoons) and interactive options (e.g., games, educational apps). However, the outcomes associated with these devices were mixed. When used passively, tablets and smartphones were associated with lower language and motor skill outcomes [26]. When apps or e-books were used with parental involvement, children demonstrated improved early literacy and language skills, indicating that the quality of content and engagement matter more than screen time alone [30]. As noted by Chen et al. [25] and Gath et al. [27], computers, though less commonly used, were generally associated with more structured and purposeful learning activities and modestly correlated with improved early literacy skills. This effect was attributed to the fact that computer-based tasks often require visual coordination and manual input and frequently involve direct parental guidance, thereby promoting parent-child interaction [25, 27, 30]. Furthermore, educational materials, particularly books and DVDs, are often accessed via computers, which may contribute to the strong self-reading habits observed in children whose only screen exposure came through computers [25]. However, their use was also more limited overall, especially among younger children, due to usability constraints like mouse and keyboard navigation. Consequently, their influence on screen time patterns and developmental outcomes in early childhood is less pervasive and more context-dependent than more portable devices.

Despite the potential educational affordances of certain devices, children with high levels of screen use, including television, tablet, smartphone, and computer use, tended to engage less in home literacy practices. This suggests that screen time, regardless of the device, may compete with traditional literacy-supportive activities in the home environment [25].

Guevara et al. [23] conducted a comparative analysis of traditional literacy practices, such as shared reading of board and print books, and digital literacy practices (using e-readers and apps) among demographically similar groups. Their study revealed that even with higher digital book usage, overall reading frequency did not increase compared to traditional board books, and that children in the digital literacy group exhibited slightly lower outcomes in language scores, verbalizations, and socially reciprocal interactions. While these differences did not show statistical significance, the authors suggested that the variation may be due to reduced interactive dialogue between parent and child and the distracting nature of multimedia features, which can shift focus away from the story, along with the physical constraints of small screen sizes. As a result, the quality of language exposure during digital reading was generally lower than with traditional books [23].

Regarding screen time measurement, six [25-30] out of the eight studies explicitly measured or reported screen time usage among their participants. The reported average daily screen time ranged from 45 minutes to approximately 103.5 minutes per day, though in some cases, children used screens for up to eight hours a day, with younger children (aged two to three years) using devices longer than older peers. This trend is often attributed to parents relying on screens to soothe or entertain their young children. Given the limited availability of structured activities and the underdeveloped play or verbal skills in this age group, screens frequently emerge as the default option for engagement. Furthermore, some parents perceive early exposure to screens as educational, which contributes to the increased screen time among younger children. These patterns underscore the complexity of digital media exposure and the varied ways in which devices shape children’s learning environments.

Grey Literature and App-Based Evidence

In this review, two studies were categorized as nontraditional evidence sources: one doctoral dissertation [26] representing grey literature and one app-evaluation study [24] that analyzed educational content rather than direct child outcomes. The dissertation offered valuable observational insights into parent-child interactions with mobile media, highlighting how engagement quality and parental self-efficacy influence learning opportunities. However, as a single, unpublished work with limited peer review, its findings are to be interpreted cautiously. The app-based analysis, while methodologically distinct, provided a useful lens for evaluating the linguistic and educational quality of preschool applications and emphasized how digital design can shape language input and feedback mechanisms.

Together, these sources broaden the conceptual understanding of children’s digital learning environments, but were not weighted equally with peer-reviewed, participant-based studies. Their inclusion underscores the diversity of emerging evidence while reinforcing the need for future primary research to confirm and extend these preliminary insights.

Quality of Content

Our findings show that interactivity and content quality are crucial factors in children's screen time, potentially outweighing the sheer amount of screen usage [24, 28, 30]. Audio language, or a combination of audio and text, is particularly beneficial for young children, as it increases the number of words they hear. Since preschool-aged children have limited reading skills, this multimodal approach is preferred over screen content that relies solely on text [24].

The majority of parents recognize the educational potential of screen time but struggle to differentiate between high-quality and low-quality content. Some parents reported using e-books as a tool for reading with their children, indicating a preference for interactive and educational content [24, 25, 28].

In light of these challenges, Taylor et al. [24] propose that the presence of specific learning goals (LGs), such as problem-solving, meaningful learning, feedback, language quality, and adjustable content, can serve as a helpful guide for caregivers and educators when selecting educational applications for children. In their study of applications across multiple platforms, they found that 13 out of 18 applications with LGs contained activities aimed at language development, while none of the 12 without LGs did. Furthermore, it was more likely for applications with LGs to provide rich language input and incorporate words aligned with the early stages of language acquisition, suggesting that they better cater to young children’s developmental needs.

However, despite the advantages of apps with clear LGs, Taylor et al. [24] noted that these apps still have substantial room for improvement in areas such as fostering exploratory use, providing adjustable content, and offering meaningful feedback. Notably, many of the apps with LGs were explicitly designed to target language development, which may have contributed to the higher quality of language input provided. 

Socioeconomic and Parental Factors

Children from low SES and low-income households demonstrate higher levels of screen-based media use, particularly television [27]. This pattern has been linked to parental efficacy, defined as caregivers’ belief in their ability to foster a positive developmental environment [25]. Parents from lower SES backgrounds, characterized by limited income and educational attainment, are more likely to report reduced efficacy, greater child screen time with less adult mediation, and a lower availability of picture books in the home. These findings may stem from structural constraints such as time scarcity, financial stress, and lack of access to literacy-promoting materials or guidance, inhibiting parents’ capacity to engage in enriching, screen-free interactions with their children.

Interestingly, the number of children in a household also moderates these associations. Families with a single child reported lower screen exposure, increased frequency of parental reading and storytelling, and higher perceived efficacy, though they did not necessarily possess a greater number of books, nor did they significantly differ in fostering children's independent reading [25]. It is possible that parents with one child experienced less stress and could provide more focused attention and engagement, leading to lower screen time and more frequent literacy activities due to concentrated resources, easier monitoring, and a greater sense of responsibility.

A subset of caregivers, described as "book-parents," typically from higher SES backgrounds, exemplify strong parental efficacy and prioritize literacy-rich environments. These parents provide extensive literary resources, often over 50 picture books, and engage consistently in shared reading and storytelling. Notably, some in this group also allow substantial computer use, yet do so without compromising the volume or quality of home literacy practices, indicating that screen media is used as a complement rather than a substitute for reading [25].

Beyond socioeconomic factors, family dynamics significantly influence children's media exposure and literacy development. Children's screen habits often reflect those of their caregivers, with parental device usage serving as both a model and a predictor of child behavior [25-27]. Chaibal et al. [29] reported a positive correlation between children's screen time and that of their mothers and other caregivers, highlighting the importance of parental habits and involvement. For instance, when mothers and relatives use screens for extended periods, children are likely to do the same, suggesting that caregiver media behavior is crucial in shaping children's screen habits. Additionally, caregivers frequently use screen time as a practical tool for distraction or soothing, especially when they want to minimize interruptions [25]. Therefore, interventions aimed at reducing screen time in early childhood must also focus on modifying parental media habits.

Researchers consistently emphasize the importance of promoting high-quality, educational, and interactive media experiences, integrated with frequent parent-child engagement. This dual approach supports healthy cognitive, linguistic, and social development, offering a more nuanced alternative to blanket screen-time restrictions [23, 25, 27, 28, 30].

Newer Trends in Screen Devices

Recently developed preschool "smart" toys have integrated screen-based and interactive media technologies, reshaping the definition of screen devices [30]. These products commonly claim to offer supportive environments where artificial intelligence can provide education, entertainment, and companionship [25]. While not designed to replace parents, their impact on parent-child interaction remains largely unstudied. If used primarily to alleviate parental anxiety or serve as digital babysitters, much like televisions, the educational potential of these high-tech toys is unlikely to be fully realized [30].

Comparison With Other Evidence

In our systematic review, we sought to evaluate the impact of screen time on language development and vocabulary acquisition in early childhood. Our findings align with a growing body of literature that underscores the complex relationship between screen exposure and language outcomes. Both our review and various systematic studies consistently indicate that excessive screen time, particularly from passive sources like television, correlates with negative effects on language development and communication skills. For instance, Massaroni et al. emphasize that prolonged screen time in early childhood can impair overall cognitive development, including critical communication skills [31]. Similarly, Madigan et al. demonstrate that higher quantities of screen time correlate with lower language skills, reinforcing our conclusions regarding the detrimental impacts of high screen exposure during formative years [32].

A significant theme emerging from our review is the role of interactive content in fostering vocabulary growth and enhancing language skills. We found that interactive and educational media can lead to improved language outcomes, a notion echoed by Bal et al. and Panjeti-Madan et al., who assert that interactive content, when aligned with recommended guidelines, can positively influence language development [7, 33]. Notably, the work of Jing et al. further supports this perspective, revealing a weak positive correlation between screen media exposure and vocabulary development, especially for e-books [34]. This meta-analysis highlights that e-books, due to their interactive nature, can significantly enhance vocabulary acquisition compared to other forms of media.

Moreover, our review specifically addresses the significance of parental involvement during screen time, a sentiment echoed in the literature. Bustamante et al. [12] and Panjeti-Madan et al. [7] support the finding that parental engagement can mitigate the negative effects of screen time, supported by findings indicating that high-quality interactions during screen activities enhance language development. Further, a systematic review by Bal et al. supports the notion that parents need to regulate their own screen time besides that of their children to increase parent-child interaction [33]. This aspect is particularly crucial, as our review notes that parental efficacy and home literacy practices significantly influence children's language outcomes.

Clinical Implications

The findings of this systematic review have important implications for clinical practice and parental guidance. Healthcare providers should counsel parents on observed associations between excessive passive screen time and weaker language outcomes and emphasize the importance of limiting screen exposure and, when needed, promoting high-quality content viewing, particularly during formative years [7-9]. This counselling should also include guidance on selecting age-appropriate and educational content, as well as strategies for actively engaging with children during screen time activities. Parents will also benefit from interventions that help them manage stress and build confidence in their parenting skills, thereby reducing compensatory screen viewing. 

These findings support the recommendations from the AAP and the WHO, which advise against screen use for infants under 18 months (except for video calls) and recommend limiting recreational screen time to one hour per day of high-quality educational content for children aged two to five, ideally with caregiver participation. Emphasizing interactive, language-rich engagement, whether on or off screens, appears most consistent with the developmental principles reflected in our synthesis.

Future Research Directions

This systematic review identifies several critical avenues for future research to refine our understanding of the nuanced relationship between screen time and language development in early childhood. High-quality RCTs are needed to evaluate the effectiveness of interventions aimed at promoting responsible screen use and maximizing the language-enhancing potential of interactive media. In addition, longitudinal studies are essential to assess the long-term impact of screen time on language trajectories and to identify critical periods of vulnerability. Moreover, future research should explore the impact of different screen modalities and content types on specific language skills, such as expressive language, receptive language, and narrative abilities. These studies should be implemented in a variety of populations to ensure broader support and clinical implementations. Furthermore, app developers can use these insights to design age-appropriate, adaptable, and linguistically enriching digital content.

Strengths and Limitations

This review, conducted according to the PRISMA 2020 guidelines [18], possesses several notable strengths. The review employed a comprehensive search strategy across multiple databases to identify a broad range of relevant studies. This multi-database approach, combined with the use of tailored keywords and MeSH terms related to screen time, language development, and vocabulary acquisition, aimed to minimize the risk of publication bias and ensure a thorough exploration of the available evidence. Furthermore, the application of clear inclusion and exclusion criteria, guided by the PI/ECOT framework, ensured that the included studies were relevant to the research question, focusing specifically on the impact of screen time on language development in early childhood. The independent screening of titles and abstracts by two reviewers, facilitated by the Rayyan app [19], enhanced the objectivity and reliability of the study selection process. Finally, the use of standardized tools for quality assessment, such as the Cochrane RoB 2 tool [20], ROBINS-I [21], and the JBI Critical Appraisal Tool for Cross-Sectional Studies [22], provided a rigorous evaluation of the methodological validity of the included studies.

Despite these strengths, this systematic review is subject to certain limitations that warrant consideration. First, while a comprehensive search strategy was employed, the restriction to studies published in English within the last five years may have introduced language bias and limited the inclusion of potentially relevant research from earlier periods or non-English sources.

Second, the heterogeneity in study designs, outcome measures, and intervention details across the included studies may limit the ability to synthesize the data quantitatively and draw firm conclusions about the overall effect of screen time on language development. For instance, the measures used to assess language development varied across studies, including standardized tests, parental reports, and observational assessments, potentially affecting the comparability of the results. This variability can influence whether or not findings can be generalized broadly.

Third, the reliance on observational studies and non-randomized clinical trials increases the risk of confounding and selection bias. While the ROBINS-I tool [21] and the JBI Critical Appraisal Tool for Cross-Sectional Studies [22] were used to assess the risk of bias in these studies, the potential for residual confounding and selection bias cannot be entirely eliminated. For example, children with pre-existing language delays may be more or less likely to engage in screen time activities, confounding the relationship between screen time and language development. Fourth, the reliance on studies with a limited age range may not truly capture the real effects of high screen time on a broad range of children. Therefore, while the evidence included gives important information regarding potential pitfalls, further research is necessary. Finally, the definition and measurement of screen time varied across studies, potentially affecting the accuracy and comparability of the findings.

## Conclusions

This systematic review concludes that the relationship between screen exposure and language development in early childhood is not a simple matter of duration but is profoundly shaped by context and content. Based on the available evidence, which we rate as very low to low certainty, the findings suggest that high amounts of passive and unsupervised screen time, particularly television, are often associated with negative impacts on language skills, largely because such use can displace crucial face-to-face interactions with caregivers. However, these potential risks are not inevitable. The current body of evidence indicates that the quality of screen engagement may be a more critical determinant of outcomes than the mere amount of time spent. Specifically, when digital content is educational and interactive, and especially when parents or caregivers actively co-view and engage with the child, potential negative associations appear to be mitigated. This can transform a potentially isolating activity into a shared, language-rich experience. Therefore, while the evidence is still evolving, the primary takeaway for clinical practice and parental guidance is a shift from blanket time restrictions to a more mindful approach that prioritizes high-quality, interactive content and emphasizes the irreplaceable value of parental involvement in a child's digital world. Future high-quality research is essential to strengthen these conclusions and provide more definitive guidance.
